# Hyperammonemia-Associated Stroke-Like Episodes and Acute Liver Failure in an 11-Month-Old Infant With Probable Ornithine Transcarbamylase Deficiency: Diagnostic and Therapeutic Challenges in a Resource-Limited Setting

**DOI:** 10.7759/cureus.104396

**Published:** 2026-02-27

**Authors:** Hind Zahiri, Fatimazahrae Naimi, Aziza Elouali, Abdeladim Babakhouya, Maria Rkain

**Affiliations:** 1 Pediatric Medicine, Mohammed VI University Hospital, Faculty of Medicine and Pharmacy, Mohammed First University, Oujda, MAR; 2 Pediatrics, Mohammed VI University Hospital, Faculty of Medicine and Pharmacy, Mohammed First University, Oujda, MAR; 3 Pediatrics, Faculty of Medicine and Pharmacy, Mohammed First University, Oujda, MAR; 4 Pediatric Gastroenterology, Hospital Center University Mohammed VI, Oujda, MAR

**Keywords:** hyperammonemia, metabolic stroke, ornithine transcarbamylase deficiency, pediatric acute liver failure (palf), stroke-like episodes, urea cycle disorders

## Abstract

Hyperammonemia outside the neonatal period is a time-critical emergency and may reveal a late-onset urea cycle disorder, including ornithine transcarbamylase deficiency (OTCD), which can be particularly challenging to recognize in females. We report the case of an 11-month-old female who presented with seizures and transient focal neurological deficits in the context of recurrent vomiting and acute hepatic dysfunction, with respiratory alkalosis and marked hyperammonemia accompanied by biochemical features compatible with a proximal urea cycle defect. Brain imaging showed stroke-like changes that were not consistent with a vascular territory, supporting a metabolic mechanism rather than primary ischemia. In a resource-limited setting where standard nitrogen-scavenging therapy and extracorporeal ammonia removal were not accessible, prompt catabolic control using strict protein restriction and high-calorie hydration was associated with biochemical recovery and clinical improvement, although mild residual weakness remained. This report highlights that early suspicion and immediate supportive management can be lifesaving when advanced diagnostics and targeted therapies are unavailable.

## Introduction

Hyperammonemia in pediatric patients may have serious outcomes, including cerebral edema, profound neurological deficits, and, in some cases, death. Pediatricians should be familiar with its clinical presentation, urgent treatment, and long-term management. In infants and children, the main differential diagnosis includes inborn errors of metabolism (IEMs), particularly urea cycle disorders (UCDs) [1,2].

Ornithine transcarbamylase deficiency (OTCD) is the most common X-linked UCD. While hemizygous males typically present with catastrophic neonatal hyperammonemia, heterozygous females exhibit a wide phenotypic spectrum due to skewed X-chromosome inactivation. Approximately 20% of female carriers develop late-onset symptoms, often triggered by catabolic stressors such as infection or high protein intake. Clinically, presentations may include acute encephalopathy, hyperventilation, seizures, or later-onset manifestations such as protein aversion, recurrent vomiting, and behavioural changes during metabolic stress [3].

Neurologic involvement in OTCD and other neurometabolic disorders may include focal deficits, encephalopathy, and seizures, sometimes with stroke-like MRI lesions that do not respect vascular territories. Hyperammonemia promotes neurotoxicity by impairing cerebral energy metabolism (via inhibition of gamma-ketoglutarate dehydrogenase) and triggering N-methyl-D-aspartate (NMDA) receptor-mediated excitotoxicity, leading to oxidative stress and cytotoxic injury; these mechanisms likely contribute to non-territorial stroke-like lesions on MRI [4].

Practical diagnostic clues include unexplained respiratory alkalosis due to hyperventilation, low urea, and hyperammonemia; in OTCD, elevated urinary orotic acid and plasma amino-acid patterns (typically increased glutamine with low citrulline/arginine) further support the diagnosis [3]. Current guidelines for managing UCDs provide well-defined approaches that include both approved and off-label therapies; however, real-world implementation may be limited by restricted access to essential treatments [5].

We report the case of an 11-month-old female infant with stroke-like episodes (SLEs), seizures, and acute hepatocellular injury in whom the association of hyperammonemia, low urea, respiratory alkalosis, and markedly increased urinary orotic acid raised a strong suspicion for a proximal UCD, most likely OTCD. However, confirmatory plasma amino acid profiling and genetic testing were not available, and the diagnosis remained probable rather than confirmed. This report highlights practical diagnostic and therapeutic challenges and emphasizes an approach based on early suspicion and rapid supportive measures in resource-limited settings.

## Case presentation

**Figure 1:**
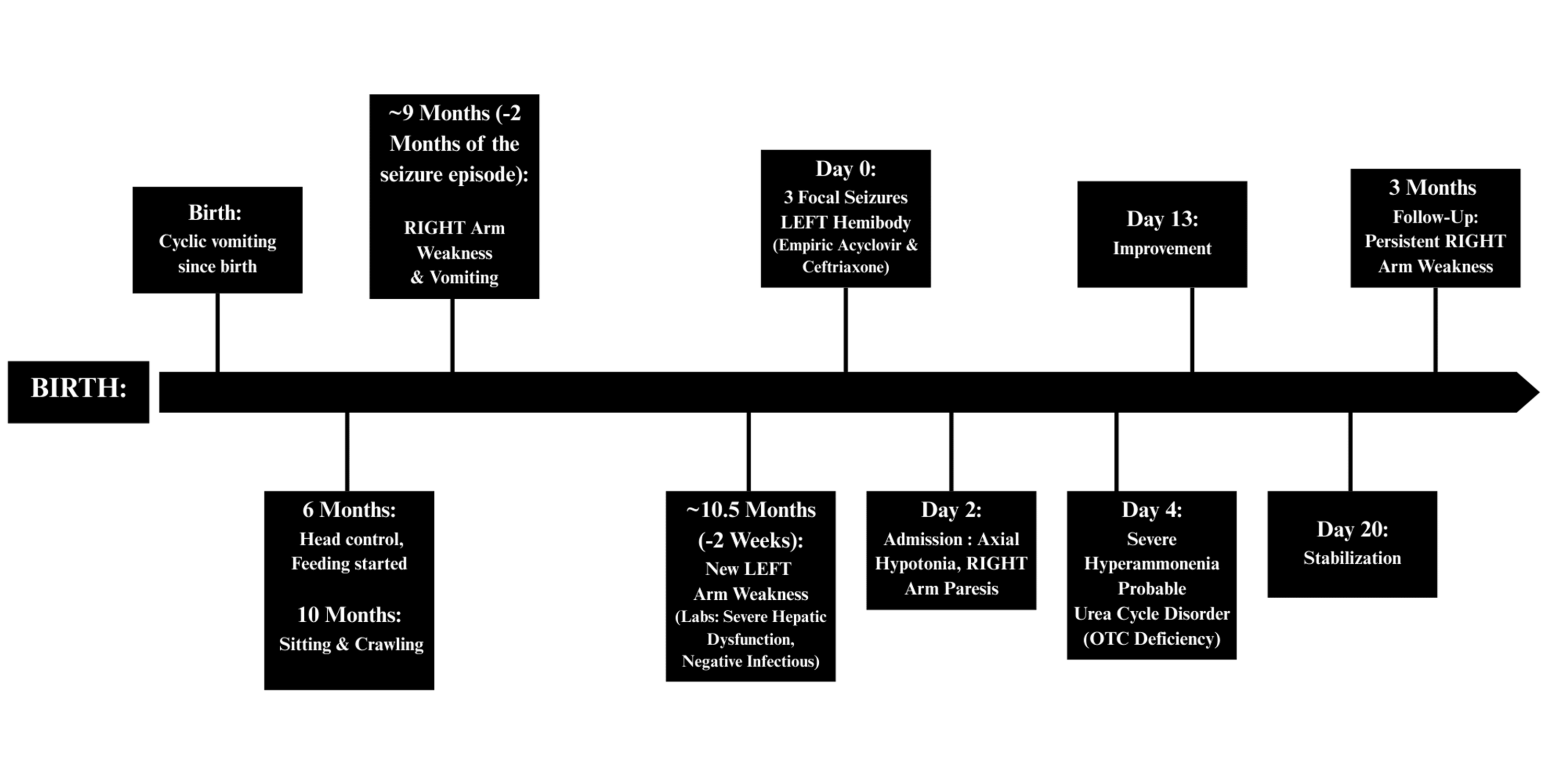
Chronological clinical timeline Timeline summarizing the clinical course of an 11‑month‑old female infant with fluctuating focal neurological deficits, acute hyperammonemic crisis, and probable ornithine transcarbamylase (OTC) deficiency.

We present the case of an 11-month-old female infant, born to non-consanguineous parents, referred to our tertiary care center for the management of afebrile focal motor seizures (Figure 1). Pregnancy, delivery, and neonatal adaptation were uneventful. Developmental milestones were moderately delayed, with head control at six months, independent sitting and crawling at 10 months, and first syllables around 11 months. The medical history was notable for cyclic vomiting since birth. Complementary feeding was introduced at six months of age. Family history included the death of a male sibling on day 7 of life from an undocumented condition.

The onset of the current illness occurred two months before the seizure episode, triggered by post-vaccination fever. This episode manifested as an abrupt right upper-limb monoparesis associated with vomiting and constipation, with spontaneous recovery within a few days. Two weeks before the seizure episode, the patient developed a new contralateral deficit (left upper-limb monoparesis). This was followed by three episodes of focal motor seizures involving the left hemibody with facial involvement within a 24-hour period (day 0). Initial management at a regional hospital included empiric acyclovir and ceftriaxone for suspected meningoencephalitis, followed by transfer to our center.

On admission (day 2 post-seizure onset), the patient was afebrile and tachypneic, with a respiratory rate of 50 breaths per minute. She was hemodynamically stable, with decreased interaction. Her weight was 10 kg, and her head circumference was 46.5 cm (within normal limits). No jaundice or dysmorphic features were noted. Neurological examination showed marked axial hypotonia and right upper-limb monoparesis. Cranial nerve examination was intact.

Initial laboratory investigations (Table 1) revealed no major electrolyte abnormalities. However, the blood urea nitrogen (BUN) was low, and the serum creatinine was also low. Arterial blood gas analysis (on room air) demonstrated uncompensated respiratory alkalosis. Liver function tests indicated severe hepatocellular injury, with markedly elevated transaminases and impaired synthetic function. Total bilirubin levels were normal.

**Table 1:** Initial laboratory work-up Units and reference ranges are reported as provided by the laboratory. ANC: absolute neutrophil count; ALT: alanine aminotransferase; AST: aspartate aminotransferase; aPTT: activated partial thromboplastin time; CMV: cytomegalovirus; CPK: creatine phosphokinase (creatine kinase); CRP: C-reactive protein; CSF: cerebrospinal fluid; EBV: Epstein-Barr virus; ESR: erythrocyte sedimentation rate; GGT: gamma-glutamyl transferase; HBsAg: hepatitis B surface antigen; anti-HBs: antibody to hepatitis B surface antigen; anti-HBc: antibody to hepatitis B core antigen; anti-HCV: antibody to hepatitis C virus; HIV: human immunodeficiency virus; Ag: antigen; Ab: antibody; p24: HIV p24 antigen; anti-HAV: antibody to hepatitis A virus; HSV: herpes simplex virus; INR: international normalized ratio; LDH: lactate dehydrogenase; MCV: mean corpuscular volume; MCH: mean corpuscular hemoglobin; MCHC: mean corpuscular hemoglobin concentration; PaCO_2_: arterial partial pressure of carbon dioxide; HCO_3_⁻: bicarbonate; PaO_2_: arterial partial pressure of oxygen; SaO_2_: arterial oxygen saturation; PCR: polymerase chain reaction; PT: prothrombin time; VCA: viral capsid antigen; WBC: white blood cell count

Parameter	Result	Reference range	Interpretation
Blood glucose	0.8 g/L	0.50-0.99 g/L	Normal
Calcium (infant)	89 mg/L	62-110 mg/L	Normal
Total protein	45 g/L	56-75 g/L	Low
Potassium	4.7 mEq/L	4.1-5.3 mEq/L	Normal
Sodium	140 mEq/L	139-146 mEq/L	Normal
Urea	0.08 g/L	0.10-0.30 g/L	Low
Creatinine (kinetic Jaffe)	3.11 mg/L	5.7-11.1 mg/L	Low
Creatine phosphokinase (CPK)	136 IU/L	29-168 IU/L	Normal
White blood cells	11,080/µL	4,000-10,000/µL	Mildly high
Hemoglobin	13.3 g/dL	12-16 g/dL	Normal
MCV	86.5 fL	80-98 fL	Normal
MCH	30.4 pg	27-32 pg	Normal
MCHC	35.1%	32-36%	Normal
Platelets	315,000/µL	150,000-400,000/µL	Normal
Neutrophils (ANC)	8,110/µL	1,500-7,000/µL	High
Lymphocytes	2,190/µL	1,000-4,000/µL	Normal
Monocytes	740/µL	200-800/µL	Normal
Eosinophils	20/µL	0-500/µL	Normal
Basophils	20/µL	0-200/µL	Normal
ESR	9 mm	-	Low/normal
CRP	0.34 mg/L	0-5 mg/L	Normal
Lumbar puncture (CSF)	Clear; no pleocytosis (1 WBC/mm^3^)	No pleocytosis	Normal
HSV PCR (CSF)	Negative	-	Negative
HBsAg (quantitative)	0.00 IU/mL	Negative < 0.05	Negative
Anti-HBs	101.50 mIU/mL	Positive > 10	Immune
Anti-HBc (IgG + IgM)	0.07	Negative index < 1	Negative
Anti-HBc IgM	0.15	Negative index < 1	Negative
Anti-HCV (screening)	0.12	Negative index < 1	Negative
HIV-1/2 (Ag p24 + Ab)	0.34	Negative index < 1	Negative
Anti-HAV IgG	0.50	Negative index < 1	Negative
Anti-HAV IgM	Negative	-	Negative
CMV IgG	0.60 AU/mL	Non-reactive < 6.0	Non-reactive
CMV IgM	0.11	Non-reactive index < 0.85	Non-reactive
EBV VCA IgG	0.09	Non-reactive < 0.75	Non-reactive
EBV VCA IgM	0.04	Non-reactive < 0.50	Non-reactive
AST	430 IU/L	5-34 IU/L	Markedly high
ALT	1279 IU/L	0-55 IU/L	Markedly high
Total bilirubin	3.80 mg/L	2.00-12.00 mg/L	Normal
Direct bilirubin	2.00 mg/L	0.00-5.00 mg/L	Normal
GGT	47 IU/L	9-36 IU/L	Mildly high
LDH	250 IU/L	125-243 IU/L	Slightly high
Alkaline phosphatase	300 IU/L	<500 IU/L	Normal
Albumin	40 g/L	35-50 g/L	Normal
Prothrombin time (PT, %)	48%	70-100%	Low
INR	1.60	~0.99	High
aPTT (patient)	29.6 s	-	-
aPTT (control)	25.0 s	-	-
aPTT ratio	1.18	-	Slightly high
Coagulation factor V	57.5%	70-120%	Low
Fibrinogen	1.80 g/L	2.00-4.00 g/L	Low
pH	7.56	-	Alkalemia
PaCO2	25 mmHg	-	Hypocapnia
HCO3-	22 mmol/L	-	Metabolic compensation
Base excess	-4	-	Mild base deficit
PaO2	90 mmHg	-	Adequate
SaO2	97%	-	Adequate
Overall	-	-	Respiratory alkalosis

The infectious workup, including C-reactive protein and extensive viral serologies (Hepatitis A, B, C; cytomegalovirus; Epstein-Barr virus; HIV), was negative. Cerebrospinal fluid analysis showed no pleocytosis, and herpes simplex virus (HSV) polymerase chain reaction (PCR) was negative, leading to discontinuation of acyclovir and ceftriaxone. Abdominal ultrasonography was normal.

**Figure 2:**
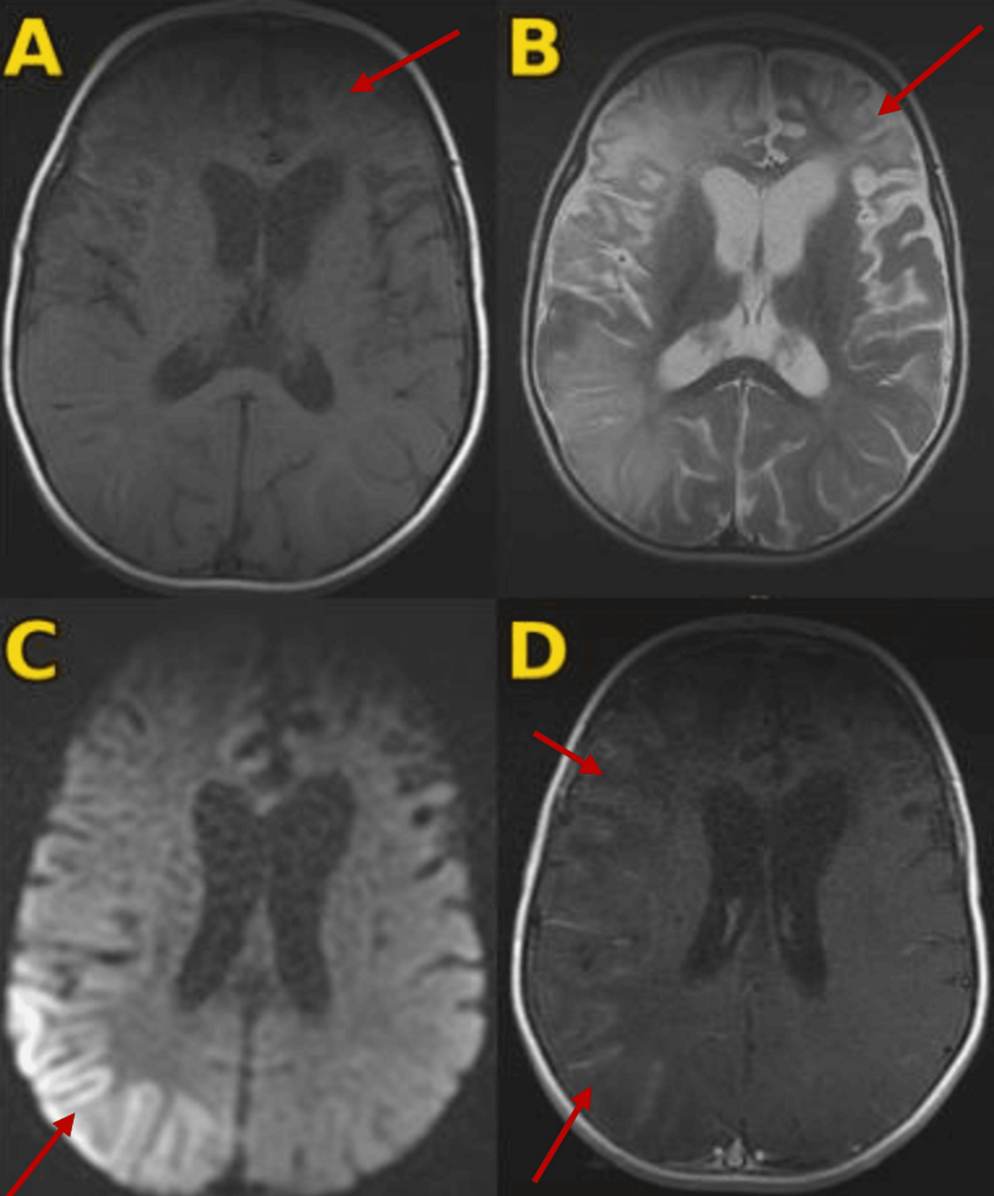
Brain MRI performed on day 3 of seizure onset A: Axial T1-weighted sequence showing a left frontal cortico-subcortical signal abnormality that is hypointense on T1, associated with enlargement of the ipsilateral frontal horn, consistent with chronic/post-injury sequelae (red arrow). B: Axial T2-weighted sequence showing the same left frontal cortico-subcortical lesion, hyperintense on T2, with ex vacuo dilatation of the ipsilateral frontal horn (red arrow). C: Diffusion-weighted imaging (DWI) demonstrating marked diffusion hyperintensity in the right parietal cortico-subcortical region involving the cortex and adjacent subcortical white matter; the pattern is non-territorial and does not conform to a specific arterial vascular territory (red arrow). D: Axial post-contrast T1-weighted image showing marked leptomeningeal enhancement in the right fronto-temporo-parietal region (red arrows).

Brain MRI performed on day 3 of seizure onset (Figure 2) revealed a chronic-appearing lesion involving the left frontal cortex and subcortical white matter (T2 hyperintense, T1 hypointense) without diffusion restriction, associated with ex vacuo dilatation of the ipsilateral frontal horn; diffusion-weighted imaging (DWI) demonstrating marked diffusion hyperintensity in the right parietal cortico-subcortical region involving the cortex and adjacent subcortical white matter; and right fronto-parieto-temporal leptomeningeal enhancement without hemorrhagic changes. The overall pattern did not conform to a single arterial territory, making a typical territorial arterial ischemic stroke less likely. MR angiography was not performed, representing a limitation. Standard electroencephalography (EEG) showed right fronto-central asymmetry without epileptiform discharges.

Given the association of neurological manifestations and acute hepatocellular injury with impaired synthetic function, toxic and infectious causes were considered. No exposure to hepatotoxic drugs, including acetaminophen, was reported, and infectious etiologies were excluded (Table 1). Pediatric acute liver failure (PALF) criteria were considered; however, the presentation did not meet PALF criteria (international normalized ratio (INR) remained <2.0, and the encephalopathy could not be confidently attributed to liver failure). In view of the afebrile presentation, fluctuating neurological episodes, cyclic vomiting, and a history of neonatal death in a male sibling, an inborn error of metabolism was prioritized.

First-line metabolic testing (Table 2) performed on day 4 of seizures revealed severe hyperammonemia at 251 µmol/L (reference 18-72.2 µmol/L), with normal lactate, normal creatine phosphokinase (CPK), and negative ketonuria (blood ketones were unavailable). In the absence of hyperlactatemia, the combination of hyperammonemia, low urea, respiratory alkalosis, and anicteric liver involvement suggested a UCD. The female sex and neonatal death in a male sibling raised suspicion for X-linked OTCD. Plasma amino acid analysis was not available due to resource limitations. Other inherited metabolic disorders (IMDs) were also considered. Organic acidemias and mitochondrial disorders commonly present with metabolic acidosis and/or hyperlactatemia, which were not observed in our patient (normal lactate and no metabolic acidosis). Fatty acid oxidation defects typically show hypoketotic hypoglycemia during decompensation; ketonuria was negative, and glucose was not suggestive of this pattern. Urinary orotic acid, obtained subsequently, was markedly elevated: 768.3 µmol/L; 1113.5 mmol/mol creatinine (reference: 0.3-4.1 mmol/mol creatinine), supporting a proximal urea cycle defect (specifically OTCD). Genetic confirmation could not be obtained.

**Table 2:** First-line metabolic tests CPK: creatine phosphokinase (creatine kinase, CK); IU: international unit(s); mmol/L: millimoles per liter; µmol/L: micromoles per liter

Parameter	Result	Reference range	Interpretation
Plasma ammonia	251 µmol/L	18-72.2 µmol/L	Severely elevated
Lactate	1.33 mmol/L	0.5-2.2 mmol/L	Normal
CPK	136 IU/L	29-168 IU/L	Normal
Ketone bodies (blood)	Not performed	-	Not available

Once hyperammonemia was identified (day 5 of seizures), emergency management was initiated, including cessation of protein intake, high-calorie intravenous hydration to prevent catabolism, and vitamin K supplementation. Seizures were treated with levetiracetam (titrated to 50 mg/kg/day). Sodium valproate was avoided, given its contraindication in suspected UCDs. Ammonia scavengers (sodium benzoate/phenylbutyrate) and extracorporeal ammonia removal were unavailable.

Plasma ammonia decreased to 117 µmol/L by day 7, and to 52 µmol/L by day 13 post seizure. Prothrombin time remained <50% until day 13, then improved to 85%, in parallel with improvement of transaminases (aspartate aminotransferase (AST) 115 IU/L; alanine aminotransferase (ALT) 198 IU/L on day 13). At the three-month follow-up, the patient had persistent right upper-limb weakness with hyperreflexia. Long-term management included a protein-restricted diet, multivitamin supplementation, and multidisciplinary rehabilitation.

## Discussion

Ammonia arises from amino acid catabolism, glutamine dehydrogenase activity in the liver, kidneys, pancreas, and brain, and from adenosine monophosphate (AMP) deamination in skeletal muscle during exercise. Most of the ammonia generated is processed in hepatocytes via the urea cycle and excreted in urine as urea, or detoxified through conversion to glutamine, part of which is subsequently eliminated by the kidneys [2]. In its non-ionized form, ammonia readily crosses the blood-brain barrier and can accumulate markedly in the brain during severe hyperammonemia. Elevated ammonia levels are profoundly neurotoxic and may cause tremor, ataxia, seizures, coma, and, in extreme cases, death [6]. Hyperammonemia is classically attributed to primary defects of urea cycle enzymes, secondary disturbances in related pathways that indirectly impair urea cycle function, or other conditions that increase ammonia production or reduce its clearance, including hepatic dysfunction, infections, medications, and transient neonatal states. Most primary and secondary etiologies fall within IMDs, also referred to as IEMs, which can be rapidly fatal if unrecognized and untreated [7]. In our patient, the association of acute liver failure (ALF) with encephalopathy initially suggested infectious or toxic causes, which are frequent etiologies of fulminant hepatic failure in infancy; however, several clinical and biochemical features made these diagnoses less likely.

UCDs are rare IMDs in which impaired ureagenesis limits ammonia elimination; in the “classic” UCDs, the defect involves a core cycle enzyme, including carbamoyl phosphate synthetase 1 deficiency, OTCD, argininosuccinate synthetase 1 deficiency, argininosuccinate lyase deficiency, and arginase 1 deficiency [8]. Neonates with minimal residual activity of these proteins, particularly defects involving the first four urea cycle enzymes or the cofactor-producing enzyme N-acetylglutamate synthetase (NAGS), may appear well at birth but typically deteriorate within the first days of life with hyperammonemia, whereas partial deficiencies can remain silent for years until a stressor overwhelms limited ureagenesis capacity and precipitates a hyperammonemic crisis [9]. UCD subtypes can be oriented by characteristic biochemical patterns combining ammonia levels, key amino acids, and urinary orotic acid (Table 3), which guide the initial differential diagnosis and selection of confirmatory testing; definitive confirmation is most often achieved by targeted molecular genetic analysis [5]. OTCD is an X-linked disorder and the most prevalent UCD [10]. Severely affected males with neonatal-onset OTCD are usually well at birth but develop symptoms within the first week, often around days 2-3, and may already be critically ill at presentation; recurrence after an initial neonatal hyperammonemic coma is common despite appropriate therapy, and many ultimately require liver transplantation. In contrast, males and heterozygous females with post-neonatal (partial) OTCD may present at any age from infancy to adulthood [3].

**Table 3:** Urea cycle disorders - diagnostic summary Information summarized from Häberle et al. [5]. NAGS: N-acetylglutamate synthase; CPS1: carbamoyl phosphate synthetase 1; OTC: ornithine transcarbamylase; ASS1: argininosuccinate synthetase 1; ASL: argininosuccinate lyase; ARG1: arginase 1 (genes/enzymes); ASA: argininosuccinic acid; HHH: hyperornithinemia-hyperammonemia-homocitrullinuria

Disorder	Key biochemical pattern	Urinary orotic acid	Confirmation/key notes
NAGS/CPS1 deficiency	Hyperammonemia; glutamine high; citrulline and arginine low.	Normal	Genetic testing (NAGS, CPS1). Carbamylglutamate response suggests NAGS (not definitive).
OTC deficiency	Hyperammonemia; glutamine and alanine high; citrulline low.	Very high	OTC gene testing (X-linked). If inconclusive: enzyme activity (plasma/liver/intestinal mucosa) or allopurinol load test (carriers).
ASS1 deficiency (Citrullinemia type 1)	Hyperammonemia; citrulline very high; ASA absent.	Increased	ASS1 gene testing (also supports prenatal testing; helps distinguish from citrullinemia type 2).
ASL deficiency (Argininosuccinic aciduria)	ASA markedly high in plasma/urine; citrulline often elevated.	Often increased	ASL gene testing (prognosis/prenatal). Enzyme assay possible (fibroblasts/RBCs), but metabolites + genetics are standard.
Arginase 1 deficiency (Argininemia)	Arginine high (often >300 umol/L); hyperammonemia less frequent.	Increased	ARG1 gene testing or erythrocyte arginase activity. Often later onset with progressive spastic paraplegia.
HHH syndrome (ORNT1 deficiency)	Triad: hyperornithinemia, hyperammonemia, homocitrullinuria.	Often increased	SLC25A15 (ORNT1) gene testing or fibroblast functional testing (labeled ornithine incorporation). Can include slowly progressive spastic paraparesis.

In females, clinical variability is largely driven by hepatic X-chromosome inactivation (lyonization), with the liver forming a mosaic of hepatocyte clusters expressing either the wild-type or mutant allele, as shown by Cunningham et al. (2023) [11]. Although genetic confirmation was not available, the association of hyperammonemia with orotic aciduria strongly supports OTCD in our patient and offers a unifying explanation for discordant sibling outcomes: the male sibling’s early death is consistent with a likely fatal neonatal hemizygous form, whereas our patient’s course is compatible with a symptomatic heterozygous carrier state modulated by lyonization.

Hepatic involvement is increasingly recognized in OTCD and may affect more than half of symptomatic patients, ranging from mild transaminase elevation to ALF that can represent the initial presentation or recur during hyperammonemic episodes [12]. While ALF is well described in severely affected male neonates, recent cohort data underscore substantial risk in late-onset disease, with ALF reported in four of five late-onset patients (predominantly females) compared with one early-onset male [13].

Urea cycle dysfunction has also been associated with stroke and SLEs, reported in CPS1 deficiency, OTCD, and citrullinemia, although mechanisms remain incompletely understood; hyperammonemia may contribute through disrupted amino acid metabolism, altered neurotransmission, and oxidative stress, culminating in neurotoxicity [4]. In our patient, this metabolic neurotoxicity likely explains the recurrent hemiparesis and focal seizures, supported by MRI diffusion restriction in non-vascular territories (left frontoparietal and temporo-insular regions), favouring ammonia-related cytotoxic edema over thromboembolic occlusion and temporally coinciding with peak hepatocellular injury and hyperammonemia.

Management of UCDs is typically structured around acute decompensation treatment and long-term care. In acute crisis, the priority is rapid ammonia clearance using intravenous nitrogen-scavenging agents (sodium benzoate alone or combined with sodium phenylacetate) alongside intravenous arginine hydrochloride to correct deficiencies, support residual cycle flux, and stimulate CPS1 activity; adequate non-protein caloric intake is essential to promote anabolism, while protein restriction should be brief (generally 24-48 hours) to limit catabolism. Extracorporeal ammonia removal should be considered early in severe hyperammonemia, particularly in the presence of significant encephalopathy, seizures/coma, rapidly rising ammonia, or insufficient decline despite initial medical therapy, as thresholds and urgency depend on clinical severity and response rather than a single cutoff [8,4]. For chronic management, oral nitrogen scavengers such as sodium benzoate and/or sodium or glycerol phenylbutyrate reduce nitrogen burden, and in OTCD, citrulline supplementation may provide additional benefit [8]. Despite clear guideline-based strategies, real-world implementation can be hindered by limited access to essential therapies; a survey of UCD care in Germany and Austria reported that emergency treatments were unavailable in 34% of participating hospitals [14]. Our case illustrates these challenges in a resource-limited setting where first-line intravenous scavengers (sodium benzoate or phenylbutyrate) were not available, necessitating an intensive conservative approach focused on immediate protein cessation and high-calorie intravenous hydration to reverse catabolism. The favourable stabilization observed suggests that, when advanced therapies are inaccessible, strict dietary measures and catabolic control can still be effective in selected resource-constrained contexts.

## Conclusions

This case illustrates the devastating potential of hyperammonemia in infancy, manifesting here as a complex interplay of metabolic stroke and hepatocellular injury. While the biochemical profile, particularly orotic aciduria, pointed towards a probable diagnosis of OTCD, the unavailability of confirmatory testing highlights the diagnostic hurdles faced in low-resource environments. Importantly, our experience suggests that early supportive “metabolic resuscitation” (brief protein cessation and provision of adequate non-protein calories to reverse catabolism) can contribute to initial clinical and biochemical stabilization when guideline-recommended therapies (intravenous nitrogen scavengers and extracorporeal ammonia removal) are unavailable. Whenever possible, management should follow established guidelines and include timely access to definitive diagnostics and targeted therapies to minimize neurological sequelae.
